# Supplementary material for: Down regulation of vestibular balance stabilizing mechanisms to enable transition between motor states
Source: eLife. 2018 Jul 10;7:e36123. doi: 10.7554/eLife.36123 (PMC6056236; doi:10.7554/eLife.36123)
Supplement: Transparent reporting form [file elife-36123-transrepform.docx]

***eLife’s* transparent reporting form**

We encourage authors to provide detailed information *within their submission* to facilitate the interpretation and replication of experiments. Authors can upload supporting documentation to indicate the use of appropriate reporting guidelines for health-related research (see [EQUATOR Network](http://www.equator-network.org/%20)), life science research (see the [BioSharing Information Resource](https://biosharing.org/)), or the [ARRIVE guidelines](http://www.plosbiology.org/article/info:doi/10.1371/journal.pbio.1000412) for reporting work involving animal research. Where applicable, authors should refer to any relevant reporting standards documents in this form.

If you have any questions, please consult our Journal Policies and/or contact us: [editorial@elifesciences.org](mailto:editorial@elifesciences.org).

**Sample-size estimation**

- You should state whether an appropriate sample size was computed when the study was being designed
- You should state the statistical method of sample size computation and any required assumptions
- If no explicit power analysis was used, you should describe how you decided what sample (replicate) size (number) to use

Please outline where this information can be found within the submission (e.g., sections or figure legends), or explain why this information doesn’t apply to your submission:

Our sample-size estimation was based on previously published work from our research group (Dakin et al. 2007; Blouin et al. 2011; Luu et al. 2012; Dakin et al. 2013; Forbes et al. 2016). We have demonstrated repeatedly that a sample of 8 to 10 human subjects provides sufficient power to discriminate coherence values that are different from zero. Given the robustness of the observed results, we limited our analysis to 10 human subjects in the locomotor transition experiment and 6 humans subjects in the posture-to-posture experiment (control experiment).

The number of subjects can be found in the first paragraph of both the Results and the Methods sections. In addition, references to all our previous studies can be found in the current manuscript.

References:

Dakin CJ, Son GML, Inglis JT, Blouin J-S. Frequency response of human vestibular reflexes characterized by stochastic stimuli. J Physiol 583: 1117–1127, 2007

Blouin J-S, Dakin CJ, van den Doel K, Chua R, McFadyen BJ, Inglis JT. Extracting phase-dependent human vestibular reflexes during locomotion using both time and frequency correlation approaches. J Appl Physiol 111: 1484–1490, 2011

Luu BL, Inglis JT, Huryn TP, Van der Loos HFM, Croft EA, Blouin J-S. Human standing is modified by an unconscious integration of congruent sensory and motor signals. J Physiol 590: 5783–94, 2012

Dakin CJ, Inglis JT, Chua R, Blouin J-S. Muscle-specific modulation of vestibular reflexes with increased locomotor velocity and cadence. J Neurophysiol 110: 86–94, 2013

Forbes PA, Luu BL, Van der Loos HFM, Croft EA, Inglis JT, Blouin JS. Transformation of vestibular signals for the control of standing in humans. J Neurosci 36: 11510–11520, 2016

**Replicates**

- You should report how often each experiment was performed
- You should include a definition of biological versus technical replication
- The data obtained should be provided and sufficient information should be provided to indicate the number of independent biological and/or technical replicates
- If you encountered any outliers, you should describe how these were handled
- Criteria for exclusion/inclusion of data should be clearly stated
- High-throughput sequence data should be uploaded before submission, with a private link for reviewers provided (these are available from both GEO and ArrayExpress)

Please outline where this information can be found within the submission (e.g., sections or figure legends), or explain why this information doesn’t apply to your submission:

We report the results of two experiment. The first experiment (locomotor transition) was performed once for each of the ten participants. Thus, because we included 10 participants, this experiment was repeated 10 times (once on each subject). The second experiment (posture-to-posture) was performed once for each of the six participants. Thus, because we included 6 participants, this experiment was repeated 6 times (once on each subject). This information is provided in the Material & Methods section.

Biological replicates are when the same type of organism is grown/treated under the same conditions. However, because we tested only human subjects, and each subject was only tested once, we did not define biological replicates in our paper. We did our best to control the variability in our sample by including only university-aged adults, with a small variability in age, height and weight and no known balance nor vestibular deficits. This information is provided in the Material & Methods section, header “Population”, lines 307-315.

A technical replicate is the same sample tested multiple times. As stated previously, we did not replicate experiments on the same sample nor on any of the subjects. Each subject was only tested once in each experimental condition.

We did not encounter any outliers. The results are robust: we report results from individual subjects when appropriate to illustrate how robust the results are.

In the locomotor transition experiment, trial data were excluded only if we observed one of the following: subjects (i) did not use the same foot to initiate locomotion, (ii) did not use the same foot to terminate locomotion, (iii) terminated locomotion with part of the feet outside of the forceplates or (iv) changed their head position before the end of the trial while being on the forceplates. In the posture-to-posture experiment, trial data were excluded only if we observed one of the following: subjects (i) did not shift their weight to their preferred leg, (ii) stabilized themselves with a load on the preferred leg more or less than the preferred range of 85-95% of body weight, or (iii) changed their head position before the end of the trial. Such trials were not used for analysis. This is stated at the lines 393-395 (locomotor transition) and the lines 409-411 (posture-to-posture experiment) of the manuscript, in the Material & Methods section.

No DNA data sequencing was performed in our study.

**Statistical reporting**

- Statistical analysis methods should be described and justified
- Raw data should be presented in figures whenever informative to do so (typically when N per group is less than 10)
- For each experiment, you should identify the statistical tests used, exact values of N, definitions of center, methods of multiple test correction, and dispersion and precision measures (e.g., mean, median, SD, SEM, confidence intervals; and, for the major substantive results, a measure of effect size (e.g., Pearson's r, Cohen's d)
- Report exact p-values wherever possible alongside the summary statistics and 95% confidence intervals. These should be reported for all key questions and not only when the p-value is less than 0.05.

Please outline where this information can be found within the submission (e.g., sections or figure legends), or explain why this information doesn’t apply to your submission:

For statistics we used two methods. First, we used a coherence analysis, to determine whether coherence was significant, i.e. larger than a 99% confidence interval through frequencies and time. Then, to describe the differences in coherence magnitude between different movement phases, we used multivariate analysis of variance (MANOVA) with Hotelling’s T-square and paired T-test (both Bonferroni corrected) as post-hoc tests. Statistical analysis methods are described and justified in the Material & Methods section, in the header “Statistical analysis” (lines 524-558).

Raw data of the different signals recorded during one typical trial are presented in Figure 1. Details about the different signals presented can be found in the Figure 1 caption (p. 29).

As we report the results of two similar experiments in this paper, we used the same statistical procedure described above. We carefully reported the number of subjects or trials used for the computation of all results (including statistical post-hoc tests) as well as for the means ± standard deviations (for peak of coherence and frequencies) or confidence intervals (coherence results).

All p-values have been reported in the manuscript. P-values of the post-hoc T-tests are reported in the Results section (l. 101-178). P-values of the MANOVA and Hotelling’s T-square post-hoc tests are reported in Table 1 (p. 33).

For the coherence analysis, limits of the 99% confidence interval have been reported for both individual (0.045) and all-subjects (0.005 or 0.008) analyses. This information can be found in the Material & Methods section (l. 525-532) as well as in the captions of the Figures 2, 4 and 5 (p. 29-32).

(For large datasets, or papers with a very large number of statistical tests, you may upload a single table file with tests, Ns, etc., with reference to sections in the manuscript.)

**Group allocation**

- Indicate how samples were allocated into experimental groups (in the case of clinical studies, please specify allocation to treatment method); if randomization was used, please also state if restricted randomization was applied
- Indicate if masking was used during group allocation, data collection and/or data analysis

Please outline where this information can be found within the submission (e.g., sections or figure legends), or explain why this information doesn’t apply to your submission:

Ten subjects belong to the experimental group who performed the locomotor transition experiment. Six subjects belong to the experimental group who performed the posture-to-posture transition experiment. No restricted randomization was applied and no comparison was performed between the two experimental groups. Inside the locomotor transition experiment, subjects were randomly assigned to start with the block of trials where their head faced forward or with the block of trials where their head was turned over the left shoulder. This information is provided in the manuscript, lines 362-363, in the Material & Methods section, header “Experimental design”.

No masking was used during group allocation, data collection nor data analysis.

**Additional data files (“source data”)**

- We encourage you to upload relevant additional data files, such as numerical data that are represented as a graph in a figure, or as a summary table
- Where provided, these should be in the most useful format, and they can be uploaded as “Source data” files linked to a main figure or table
- Include model definition files including the full list of parameters used
- Include code used for data analysis (e.g., R, MatLab)
- Avoid stating that data files are “available upon request”

Please indicate the figures or tables for which source data files have been provided:

We did not upload any additional data files or code for data analysis because we did not request ethical approval to do so and subjects did not consent to this. If the reviewers wish to access the data or the code for data analysis, we will have to amend our ethics procedures and ask informed consent from the participants, which may take a few weeks to do.
